# Supplementary material for: Modelling the response to vaccine in non-human primates to define SARS-CoV-2 mechanistic correlates of protection
Source: eLife. 2022 Jul 8;11:e75427. doi: 10.7554/eLife.75427 (PMC9282856; doi:10.7554/eLife.75427)
Supplement: Supplementary file 1. [file elife-75427-supp1.docx]

**Supplementary file 1.** Criteria defining RBD-ACE2 binding inhibition (studies testing two-component spike nanoparticle vaccine) or neutralization measured on live cells with luciferase marker (mRNA-1273) as mechanistic correlate of protection of the effect of the vaccine on new cell infection. Studies are labelled Study 1 and Study 2 for the main and the additional studies testing two-component spike nanoparticle vaccine, respectively and mRNA-1273 for the third study evaluating several doses of mRNA-1273 vaccine.

| **Model** | | **Study 1** | **Study 1** | **Study 2** | **mRNA-1273** |
| --- | --- | --- | --- | --- | --- |
|  |  | RBD-ACE2 binding inhibition on β | RBD-ACE2 binding inhibition on β | RBD-ACE2 binding inhibition on β | Neutralization on β |
| **Type of covariates added in the model** | | **Time-varying** | **Baseline** | **Baseline** | **Baseline** |
| **Criterion 1** | Without adjustment | 793.71 | 793.71 | 427.63 | 469.13 |
| Best fits (BICc Value) | Group effect (β and δ) | 772.34 | 772.34 | 404.80 | 468.70 |
|  | Marker effect only (β) | 765.76 | 765.22 | 413.35 | 467.21 |
|  | Marker (β) and Group (δ) effects | 762.69 | 762.53 | 407.66 |  |
| **Criterion 2** | Group effect (β and δ) | **Conv*:*** -0.747** (0.255) | **Conv:** -0.747** (0.255) | **Vacc:** -6.01**** (0.8) | **10µg:** -0.267 (0.578) |
|  |  | **Conv-CD40:** -2.38*** (0.265) | **Conv-CD40:** -2.38*** (0.265) |  | **100µg:** -1.6** (0.621) |
| Group effect (Value (Sd)) | Group (β and δ) & marker (β) | **Conv*:*** *0.00428 (1.71)* | **Conv:** 0.303 (0.864) | **Vacc:** 2.31 (1.18) | **10µg:** 0.105(0.646) |
|  |  | **Conv-CD40:** 0.1 (2.79) | **Conv-CD40:** 0.362 (1.14) |  | **100µg:** 3.10-4 (0.84) |
| **Criterion 3** | Group effect (β and δ) | **β:** 65.5 % | **β:** 65.5 % | **β:** 64.9 % | **β:** 18.5 % |
| Expl. variability for β or δ (relative %^1^) |  | **δ:** 54 % | **δ:** 54 % | **δ:** 58.2 % | **δ:** 4.6% |
|  | Marker effect (β) | **β:** 87.4 % | **β:** 82.7 % | **β:** 70.8 % | **β:** 15.4 % |
|  |  | **δ:** 27.1 % | **δ:** 31.6% | **δ:** -17% | **δ:** 19.1 % |
|  | Marker (β) and Group (δ) effects | **β:** 79 % | **β:** 75.4 % | **β:** 66 % |  |
|  |  | **δ:** 56.7 % | **δ:** 65.6 % | **δ:** 69.9 % |  |
| *^1^ Relative percentage of inter-individual variability compared to variability obtained on model without any adjustment ; 100-(x*100)/ref* | | | | | |
| ** P < 0.05, **P < 0.01, ***P < 0.001, ****P<1e-4, Wald test for fold change different from 1* | | | | | |
