# Supplementary material for: Modelling the response to vaccine in non-human primates to define SARS-CoV-2 mechanistic correlates of protection
Source: eLife. 2022 Jul 8;11:e75427. doi: 10.7554/eLife.75427 (PMC9282856; doi:10.7554/eLife.75427)
Supplement: Supplementary file 2. [file elife-75427-supp2.docx]

**Supplementary file 2.** Model parameters for viral dynamics in both the nasopharynx and the trachea estimated by the model adjusted for groups of intervention.

| **Param.** | **Meaning** | **Value [95% CI]** | **Unit** |
| --- | --- | --- | --- |
| **β** | Viral infectivity in the naive group (x10^-6^) | 0.95 [0.18 ; 4.94] | (copies/ml)^-1^ day^-1^ |
|  | Fold change in the convalescent group | 0.18 [0.04 ; 0.88]** |  |
|  | Fold change in the Conv-CD40 group | 0.004 [0.001 ; 0.029]*** |  |
| **δ** | Loss rate of infected cells in the naive group | 1.04 [0.79 ; 1.37] | day^-1^ |
|  | Fold change in the convalescent group | 1.79 [1.21 ; 2.66]** |  |
|  | Fold change in the Conv-CD40 group | 1.80 [1.17 ; 2.75]** |  |
| **P^N^** | Viral production rate in the naso. (x10^3^) | 12.1 [3.15 ; 46.5] | virions.(cell.day)^-1^ |
| **P^T^** | Viral production rate in the trachea (x10^3^) | 0.92 [0.39 ; 2.13] | virions.(cell.day)^-1^ |
| **α_vlsg_** | Infected cells and sgRNA viral load ratio | 1.39 [1.01 ; 1.76] | Virions.cell^-1^ |
| **k** | Eclipse rate | 3 | day^-1^ |
| **c** | Clearance of *de novo* produced viruses | 3 | day^-1^ |
| **c_I_** | Clearance of inoculum | 20 | day^-1^ |
| **µ** | Percentage of infectious viruses | 10^-3^ |  |
| $\boldsymbol{T}_{\boldsymbol{0}}^{\boldsymbol{X,nbc}}$ | Initial number of target cells | 1.25x10^5^ (Naso.)  2.25x10^4^ (Trachea) | cells |
| $\mathbf{Inoc}_{\mathbf{0}}$ | Number of virions inoculated | 2.19x10^10^ | virions |
| **ω_β_** | SD of random effect on log_10_ β | 0.366 [0.160 ; 0.571] |  |
| **ω_δ_** | SD of random effect on δ | 0.170 [-0.089 ; 0.429] |  |
| **σ_VLn_** | SD of error model gRNA in naso. | 1.27 [1.01 ; 1.53] |  |
| **σ_VLt_** | SD of error model gRNA in trachea | 1.09 [0.90 ; 1.28] |  |
| **σ_sgVLn_** | SD of error model sgRNA in naso | 1.41 [0.97 ; 1.85] |  |
| **σ_sgVLt_** | SD of error model sgRNA in trachea | 1.62 [1.11 ; 2.13] |  |
