# Supplementary material for: Modelling the response to vaccine in non-human primates to define SARS-CoV-2 mechanistic correlates of protection
Source: eLife. 2022 Jul 8;11:e75427. doi: 10.7554/eLife.75427 (PMC9282856; doi:10.7554/eLife.75427)
Supplement: Supplementary file 3. [file elife-75427-supp3.docx]

**Supplementary file 3a.** Values of -2LL estimated on models with viral clearance (c=cI) and eclipse phase rate k fixed at different values.

| **c k** | **1** | **3** | **6** |
| --- | --- | --- | --- |
| **1** | 1285.29 | 1286.42 | 1289.54 |
| **5** | 871.38 | 864.50 | 866.80 |
| **10** | 773.74 | 764.29 | 768.18 |
| **15** | 749.67 | **738.71** | 742.12 |
| **20** | 749.44 | **738.40** | 740.98 |
| **30** | 750.00 | **739.51** | 741.34 |

**Supplementary file 3b.** Values of -2LL estimated on models with inoculum clearance cI and clearance of virus de novo produced c fixed at different values. The eclipse phase rate was fixed at k=3 day^-1^.

| **c c_I_** | **1** | **5** | **10** | **15** | **20** | **25** | **30** |
| --- | --- | --- | --- | --- | --- | --- | --- |
| **1** | 1286.42 | 873.30 | 777.21 | 753.72 | 754.14 | 754.32 | 754.75 |
| **2** | 1286.70 | 864.90 | 760.94 | 734.03 | 734.14 | 733.94 | 734.95 |
| **3** | 1286.41 | 864.58 | 760.69 | 734.71 | **733.85** | 734.87 | 734.48 |
| **4** | 1286.33 | 864.22 | 761.78 | 735.91 | 735.16 | 735.42 | 736.14 |
| **5** | 1286.38 | 864.50 | 762.69 | 737.13 | 735.85 | 736.37 | 736.69 |
| **10** | 1286.55 | 865.48 | 764.29 | 738.38 | 737.56 | 737.89 | 738.13 |
| **15** | 1286.23 | 865.12 | 764.79 | 738.71 | 737.54 | 738.17 | 738.75 |
| **20** | 1285.96 | 865.19 | 764.78 | 738.86 | 738.40 | 738.25 | 739.30 |
| **25** | 1286.28 | 864.93 | 764.97 | 739.20 | 738.05 | 738.37 | 739.34 |
| **30** | 1286.45 | 864.77 | 765.16 | 739.01 | 738.13 | 738.58 | 739.51 |
