# Supplementary material for: Modelling the response to vaccine in non-human primates to define SARS-CoV-2 mechanistic correlates of protection
Source: eLife. 2022 Jul 8;11:e75427. doi: 10.7554/eLife.75427 (PMC9282856; doi:10.7554/eLife.75427)
Supplement: Supplementary file 4. [file elife-75427-supp4.docx]

**Supplementary file 4**. Model parameters for viral dynamics in both the nasopharynx and the trachea estimated by the model with the viral infectivity adjusted for ACE2-RBD binding inhibition and the loss rate of infected cells adjusted for the group of treatment.

| **Param.** | **Meaning** | **Value [95% CI]** | **Unit** |
| --- | --- | --- | --- |
| **β** | Infection rate with ECLRBD=0 AU **(x10^-8^)** | 0.80 [0.21 ; 3.01] | (copies/ml)^-1^ day^-1^ |
|  | Fold $\Delta ECLRBD={10}^{3} \mathrm{AU}$ | 1.018 [1.012 ; 1.023] |  |
| **δ** | Loss rate of infected cells | 1.00 [0.76 ; 1.33] | day^-1^ |
|  | Fold change in the convalescent group | 1.78 [1.21 ; 2.61]** |  |
|  | Fold change in the Conv-CD40 group | 2.18 [1.24 ; 3.84]** |  |
| **P^N^** | Viral production rate in the naso. (x10^3^) | 9.87 [2.86 ; 34.06] | virions.(cell.day)^-1^ |
| **P^T^** | Viral production rate in the trachea (x10^3^) | 0.71 [0.16 ; 3.18] | virions.(cell.day)^-1^ |
| **α_vlsg_** | Infected cells and sgRNA viral load ratio | 1.33 [1.02 ; 1.74] | Virions.cell^-1^ |
| **k** | Eclipse rate | 3 | day^-1^ |
| **c** | Clearance of *de novo* produced viruses | 3 | day^-1^ |
| **c_I_** | Clearance of inoculum | 20 | day^-1^ |
| **µ** | Percentage of infectious viruses | 10^-3^ |  |
| $\boldsymbol{T}_{\boldsymbol{0}}^{\boldsymbol{X,nbc}}$ | Initial number of target cells | 1.25x10^5^ (Naso.)  2.25x10^4^ (Trachea) | cells |
| $\mathbf{Inoc}_{\mathbf{0}}$ | Number of virions inoculated | 2.19x10^10^ | virions |
| **ω_β_** | SD of random effect on log_10_ β | 0.223 [-0.059 ; 0.505] |  |
| **ω_δ_** | SD of random effect on δ | 0.196 [-0.090 ; 0.482] |  |
| **σ_VLn_** | SD of error model gRNA in naso. | 1.13 [0.91 ; 1.34] |  |
| **σ_VLt_** | SD of error model gRNA in trachea | 1.25 [1.02 ; 1.48] |  |
| **σ_sgVLn_** | SD of error model sgRNA in naso | 1.57 [1.09 ; 2.04] |  |
| **σ_sgVLt_** | SD of error model sgRNA in trachea | 1.33 [0.96 ; 1.70] |  |
