# Supplementary material for: Modelling the response to vaccine in non-human primates to define SARS-CoV-2 mechanistic correlates of protection
Source: eLife. 2022 Jul 8;11:e75427. doi: 10.7554/eLife.75427 (PMC9282856; doi:10.7554/eLife.75427)
Supplement: Transparent reporting form [file elife-75427-transrepform1.pdf]

Westbrook Centre
Milton Road
Cambridge CB4 1YG, UK

P 01223 855340
W elifesciences.org
T @elife

eLife's transparent reporting form

We encourage authors to provide detailed information *within their submission* to facilitate the interpretation and replication of experiments. Authors can upload supporting documentation to indicate the use of appropriate reporting guidelines for health-related research (see EQUATOR Network), life science research (see the BioSharing Information Resource), or the ARRIVE guidelines for reporting work involving animal research. Where applicable, authors should refer to any relevant reporting standards documents in this form.

If you have any questions, please consult our Journal Policies and/or contact us: editorial@elifesciences.org.

Sample-size estimation

- You should state whether an appropriate sample size was computed when the study was being designed
- You should state the statistical method of sample size computation and any required assumptions
- If no explicit power analysis was used, you should describe how you decided what sample (replicate) size (number) to use

Please outline where this information can be found within the submission (e.g., sections or figure legends), or explain why this information doesn't apply to your submission:

No data were generated for this study. Data from three different studies were used in this analysis, as referenced below.
For each of the three studies, only data collected from cynomolgus macaques were used and no data have excluded.
All information about sample-size estimation for the different studies can be found in their related papers and their respective reporting summary.
Information about laboratory animals can also be found in section **Materials and Methods** in subsection *Experimental model and subjects details*, as well as in the legend of Figure 1A ([data related to Marlin, 2021]) and in the legend of Figure 5A (data related to [Brouwer, 2021]).

In each figure displaying raw data or dynamics predicted by the model, sample size within each group of treatment is provided in the legend.

[1] Marlin, R., Godot, V., Cardinaud, S., Galhaut, M., Coleon, S., Zurawski, S., ... & Le Grand, R. (2021). Targeting SARS-CoV-2 receptor-binding domain to cells expressing CD40 improves protection to infection in convalescent macaques.

[2] Brouwer, P. J., Brinkkemper, M., Maisonnasse, P., Dereuddre-Bosquet, N., Grobben, M., Claireaux, M., ... & Sanders, R. W. (2021). Two-component spike nanoparticle vaccine protects macaques from SARS-CoV-2 infection. *Cell*, *184*(5), 1188-1200.

[3] Corbett, K. S., Flynn, B., Foulds, K. E., Francica, J. R., Boyoglu-Barnum, S., Werner, A. P., ... & Graham, B. S. (2020). Evaluation of the mRNA-1273 vaccine against SARS-CoV-2 in nonhuman primates. *New England Journal of Medicine*, *383*(16), 1544-1555.

eLife Sciences Publications, Ltd is a limited liability non-profit non-stock corporation incorporated in the State of Delaware, USA, with company number 5030732, and is registered in the UK with company number FC030576 and branch number BR015634 at the address Westbrook Centre, Milton Road, Cambridge CB4 1YG.

Replicates

- You should report how often each experiment was performed
- You should include a definition of biological versus technical replication
- The data obtained should be provided and sufficient information should be provided to indicate the number of independent biological and/or technical replicates
- If you encountered any outliers, you should describe how these were handled
- Criteria for exclusion/inclusion of data should be clearly stated
- High-throughput sequence data should be uploaded before submission, with a private link for reviewers provided (these are available from both GEO and ArrayExpress)

Please outline where this information can be found within the submission (e.g., sections or figure legends), or explain why this information doesn't apply to your submission:

No data were generated for this study. Data from three different studies were used in this analysis, as referenced below.
For each of the three studies, only data collected from cynomolgus macaques were used and no data have been excluded.
All information about technical replication for the different studies can be found in their related papers and their respective reporting summary.
In addition, information about materials and experimental systems can be found in section **Material and Methods** subsection *Evaluation of anti-spike, anti-RBD and neutralizing IgG antibodies* for the quantification of antibody response as well as in the legend of the Figure 3, Figure 1- figure supplement 3 and 4 (data related to [Marlin, 2021] and the Figure 5 (data related to [Brouwer, 2021]) for PCR, PCR, ELISA, ELISPOT, Luminex, ICS and (pseudo-) neutralization assays.

[1] Marlin, R., Godot, V., Cardinaud, S., Galhaut, M., Coleon, S., Zurawski, S., ... & Le Grand, R. (2021). Targeting SARS-CoV-2 receptor-binding domain to cells expressing CD40 improves protection to infection in convalescent macaques.

[2] Brouwer, P. J., Brinkkemper, M., Maisonnasse, P., Dereuddre-Bosquet, N., Grobben, M., Claireaux, M., ... & Sanders, R. W. (2021). Two-component spike nanoparticle vaccine protects macaques from SARS-CoV-2 infection. *Cell*, *184*(5), 1188-1200.

[3] Corbett, K. S., Flynn, B., Foulds, K. E., Francica, J. R., Boyoglu-Barnum, S., Werner, A. P., ... & Graham, B. S. (2020). Evaluation of the mRNA-1273 vaccine against SARS-CoV-2 in nonhuman primates. *New England Journal of Medicine*, *383*(16), 1544-1555.

eLife Sciences Publications, Ltd is a limited liability non-profit non-stock corporation incorporated in the State of Delaware, USA, with company number 5030732, and is registered in the UK with company number FC030576 and branch number BR015634 at the address Westbrook Centre, Milton Road, Cambridge CB4 1YG.

Statistical reporting

- Statistical analysis methods should be described and justified
- Raw data should be presented in figures whenever informative to do so (typically when N per group is less than 10)
- For each experiment, you should identify the statistical tests used, exact values of N, definitions of center, methods of multiple test correction, and dispersion and precision measures (e.g., mean, median, SD, SEM, confidence intervals; and, for the major substantive results, a measure of effect size (e.g., Pearson's r, Cohen's d)
- Report exact p-values wherever possible alongside the summary statistics and 95% confidence intervals. These should be reported for all key questions and not only when the p-value is less than 0.05.

Please outline where this information can be found within the submission (e.g., sections or figure legends), or explain why this information doesn't apply to your submission:

(For large datasets, or papers with a very large number of statistical tests, you may upload a single table file with tests, Ns, etc., with reference to sections in the manuscript.)

Data from three different studies were used in this analysis, as referenced below.
All statistical analysis performed on raw data (differences between groups, correlation between viral and immune markers) can be directly found in their related papers.

Statistical analysis were performed in this paper related to the proposed model-based framework evaluating mechanistic correlate of protection.
- Mechanistic models developed in the paper were estimated by Monolix® software, version 2019R1. In those models, inter-individual variability was taken into account by considering statistical models (Mixed effects model) on each model parameter, which can depend on covariates.
- Statistical significance of the group of intervention as covariate in the models were evaluated by a Wald test directly performed in Monolix and were reported in Supplementary file 1 and 2 via p-values.
- A flow chart of the algorithm implemented for an automatic selection of time-varying covariates in our mechanistic models is given in Figure 4 – figure supplement 2 and the algorithm is described in the section **Materials and Methods** in the subsection *Algorithm for automatic selection of biomarkers as CoP*. The statistical significance of the time-varying covariate tested by this algorithm was evaluated via 3 criteria: 1) the value of the Bayesian Information Criteria BICc evaluated for the model after adjustment for the time-varying covariate, 2) the statistical significance of the covariate group when the model was adjusted for the tested covariate and the group, evaluated by a Wald-test and 3) the explained variability induced by the time-varying covariate. These information can be found in the Supplementary file 1. In addition, we ensure the statistical difference from 0 of the regression coefficient related to the covariate (in the mixed-effects model) by calculating its confidence interval. These information can be found in Figure 4B and Figure 4 – figure supplement 1A via the value of the RSE (approximately lower than 50%).
- Estimation of model parameters can be found in the Supplementary 2. For each estimated parameter, the mean value and the 95% confidence interval are reported.

Additional information are provided in section **Quantification and statistical analysis**.

In each concerned figure, information about dispersion and precision measures are described in figure legends. In most cases, mean values within each group of intervention are represented by thick lines while 95% confidence intervals are displayed by shaded area or error bars.

[1] Marlin, R., Godot, V., Cardinaud, S., Galhaut, M., Coleon, S., Zurawski, S., ... & Le Grand, R. (2021). Targeting SARS-CoV-2 receptor-binding domain to cells expressing CD40 improves protection to infection in convalescent macaques.

[2] Brouwer, P. J., Brinkkemper, M., Maisonnasse, P., Dereuddre-Bosquet, N., Grobben, M., Claireaux, M., ... & Sanders, R. W. (2021). Two-component spike nanoparticle vaccine protects macaques from SARS-CoV-2 infection. *Cell*, *184*(5), 1188-1200.

[3] Corbett, K. S., Flynn, B., Foulds, K. E., Francica, J. R., Boyoglu-Barnum, S., Werner, A. P., ... & Graham, B. S. (2020). Evaluation of the mRNA-1273 vaccine against SARS-CoV-2 in nonhuman primates. *New England Journal of Medicine*, *383*(16), 1544-1555.

eLife Sciences Publications, Ltd is a limited liability non-profit non-stock corporation incorporated in the State of Delaware, USA, with company number 5030732, and is registered in the UK with company number FC030576 and branch number BR015634 at the address Westbrook Centre, Milton Road, Cambridge CB4 1YG.

Group allocation

- Indicate how samples were allocated into experimental groups (in the case of clinical studies, please specify allocation to treatment method); if randomization was used, please also state if restricted randomization was applied
- Indicate if masking was used during group allocation, data collection and/or data analysis

Please outline where this information can be found within the submission (e.g., sections or figure legends), or explain why this information doesn't apply to your submission:

No data were generated for this study. Data from three different studies were used in this analysis, as referenced below. For each of the three studies, only data collected from cynomolgus macaques were used and no data have been excluded. In addition, allocation into experimental groups were kept has defined in the studies.
- In [Marlin, 2021], the 18 Cynomolgus macaques were allocated into the three groups of treatment referred as "naïve" (n=6), "Convalescent" (Conv) (n=6) and "convalescent-vaccinated" (αCD40.RBD or Conv-CD40) (n=6) as reported in the Figure 1A and the Supplementary file 1.
- In [Brouwer, 2021] the 10 cynomolgus macaques were allocated into the two groups of treatment referred as "Naïve" (n=4) and "vaccinated" (n=6), as reported in Figure 5A and the Supplementary file 1.
- In [Corbett, 2021], animals were splitted into three group of treatment referred as "placebo", "10μg" and "100μg" as reported in the Supplementary file 1.

Group allocation and sample size were provided in the legend of each figure involving a stratification of the results according to the group of intervention.

[1] Marlin, R., Godot, V., Cardinaud, S., Galhaut, M., Coleon, S., Zurawski, S., ... & Le Grand, R. (2021). Targeting SARS-CoV-2 receptor-binding domain to cells expressing CD40 improves protection to infection in convalescent macaques.

[2] Brouwer, P. J., Brinkkemper, M., Maisonnasse, P., Dereuddre-Bosquet, N., Grobben, M., Claireaux, M., ... & Sanders, R. W. (2021). Two-component spike nanoparticle vaccine protects macaques from SARS-CoV-2 infection. *Cell*, *184*(5), 1188-1200.

[3] Corbett, K. S., Flynn, B., Foulds, K. E., Francica, J. R., Boyoglu-Barnum, S., Werner, A. P., ... & Graham, B. S. (2020). Evaluation of the mRNA-1273 vaccine against SARS-CoV-2 in nonhuman primates. *New England Journal of Medicine*, *383*(16), 1544-1555.

Additional data files ("source data")

- We encourage you to upload relevant additional data files, such as numerical data that are represented as a graph in a figure, or as a summary table
- Where provided, these should be in the most useful format, and they can be uploaded as "Source data" files linked to a main figure or table
- Include model definition files including the full list of parameters used
- Include code used for data analysis (e.g., R, MatLab)
- Avoid stating that data files are "available upon request"

Please indicate the figures or tables for which source data files have been provided:

No unique reagents were generated for this study. Data from three different studies were used in this analysis, as referenced below. For each of the three studies, only data collected from cynomolgus macaques were used. For the viral load, only data collected within Tracheal and nasal fluids were used, tour model incorporating only compartments for trachea and nasopharynx.

Data availability:
Data that support the finding of this study are provided in the source data files of this paper and gather data from:

1) [Marlin, 2021] used in this analysis, which are also directly available online in the section **Source data** of this related paper (https://www.nature.com/articles/s41467-021-25382-0#Sec17). Data from this study can also be found in the open-access repository Dryad using the following DOI: https://doi.org/10.5061/dryad.1zcrjdfv7.

2) [Brouwer, 2021] used in this analysis, which are also available from the corresponding authors of the related paper.

3) [Corbett, 2021] used in this analysis, which are also available online in the section **Supplementary Material** of the related paper, excel file labelled ("Supplementary Appendix 2")

Code availability:
The original code (mlxtran models and R) as well as model definition files including the full list of parameters used are available on github at the following link: https://github.com/sistm/SARSCoV2modelingNHP.

[Marlin, 2021] Marlin, R., Godot, V., Cardinaud, S., Galhaut, M., Coleon, S., Zurawski, S., ... & Le Grand, R. (2021). Targeting SARS-CoV-2 receptor-binding domain to cells expressing CD40 improves protection to infection in convalescent macaques.
[Brouwer, 2021] Brouwer, P. J., Brinkkemper, M., Maisonnasse, P., Dereuddre-Bosquet, N., Grobben, M., Claireaux, M., ... & Sanders, R. W. (2021). Two-component spike nanoparticle vaccine protects macaques from SARS-CoV-2 infection. *Cell*, *184*(5), 1188-1200.
[Corbett, 2021] Corbett, K. S., Flynn, B., Foulds, K. E., Francica, J. R., Boyoglu-Barnum, S., Werner, A. P., ... & Graham, B. S. (2020). Evaluation of the mRNA-1273 vaccine against SARS-CoV-2 in nonhuman primates. *New England Journal of Medicine*, *383*(16), 1544-1555.
